# The Inhibitory Potential of Thai Mango Seed Kernel Extract against Methicillin-Resistant *Staphylococcus Aureus*

**DOI:** 10.3390/molecules16086255

**Published:** 2011-07-25

**Authors:** Pimsumon Jiamboonsri, Pimolpan Pithayanukul, Rapepol Bavovada, Mullika T. Chomnawang

**Affiliations:** 1 Department of Pharmacy, Faculty of Pharmacy, Mahidol University, Bangkok 10400, Thailand; Email: Jiamboonsri_P@hotmail.co.th; 2 Department of Pharmacognosy and Pharmaceutical Botany, Faculty of Pharmaceutical Sciences, Chulalongkorn University, Bangkok 10330, Thailand; Email: rapepol@hotmail.co.th; 3 Department of Microbiology, Faculty of Pharmacy, Mahidol University, Bangkok 10400, Thailand; Email: scmtd@mahidol.ac.th

**Keywords:** *Mangifera indica* L., methicillin−resistant *Staphylococcus aureus*, pentagalloylglucopyranose, pseudomulticellular bacteria, penicillin G

## Abstract

Plant extracts are a valuable source of novel antibacterial compounds to combat pathogenic isolates of methicillin−resistant *Staphylococcus aureus* (MRSA), a global nosocomial infection. In this study, the alcoholic extract from Thai mango (*Mangifera indica* L. cv. ‘Fahlun’) seed kernel extract (MSKE) and its phenolic principles (gallic acid, methyl gallate and pentagalloylglucopyranose) demonstrated potent *in vitro* antibacterial activity against *Staphylococcus aureus* and 19 clinical MRSA isolates in studies of disc diffusion, broth microdilution and time−kill assays. Electron microscopy studies using scanning electron microscopy and transmission electron microscopy revealed impaired cell division and ultra−structural changes in bacterial cell morphology, including the thickening of cell walls, of microorganisms treated with MSKE; these damaging effects were increased with increasing concentrations of MSKE. MSKE and its phenolic principles enhanced and intensified the antibacterial activity of penicillin G against 19 clinical MRSA isolates by lowering the minimum inhibitory concentration by at least 5−fold. The major phenolic principle, pentagalloylglucopyranose, was demonstrated to be the major contributor to the antibacterial activity of MSKE. These results suggest that MSKE may potentially be useful as an alternative therapeutic agent or an adjunctive therapy along with penicillin G in the treatment of MRSA infections.

## 1. Introduction

The Gram-positive bacterium, *Staphylococcus aureus* (*S. aureus*), is an important pathogen that can cause life-threatening bacterial infections, such as pneumonia, meningitis, osteomyelitis, endocarditis and toxic shock syndrome [1]. The introduction of β-lactam antibiotics has greatly improved the prognosis of patients with severe staphylococcal infections; however, the resistance of *S. aureus* to wide spectrum β-lactam antibiotics, such as penicillin G, methicillin and oxacillin, has emerged in several countries [2]. Methicillin-resistant *Staphylococcus aureus* (MRSA) has become a worldwide public health problem and is a major cause of both nosocomial and community infections. Although the glycopeptide antibiotic, vancomycin, is considered indispensable for the treatment of MRSA infections, the first MRSA strain to acquire resistance to vancomycin was isolated from a Japanese patient in 1996, and this was followed by reports of similar resistant strains from the USA, France, Korea, South Africa, and Brazil [3]. Under these circumstances, the development of new antibacterial agents to control MRSA is urgently needed.

Plants are rich in a wide variety of secondary metabolites, including alkaloids, terpenes, flavonoids and tannins, all of which are known to possess antibacterial activity [4]. The ethanolic extract of Thai mango (*Mangifera indica* L. cv. ‘Fahlun’, Anacardiaceae) seed kernels (MSKE) contains a relatively high phenolic content of pentagalloylglucopyranose (PGG) (61.28%) and relatively smaller amounts of methyl gallate (MG) (0.68%) and gallic acid (GA) (0.44%) [5]. MSKE and its principles have been pharmacologically documented to have antioxidant, anti-tyrosinase, anti–inflammatory, and hepatoprotective activities [5,6] as well as anti-enzymatic activities against snake venom [7,8]. The objectives of this study were to investigate the inhibitory potential of MSKE and its isolated phenolic principles against MRSA and the capacity of these principles to modulate β-lactam resistance in MRSA. In addition, the effect of MSKE and PGG on the bacterial structure was observed using electron microscopy. 

## 2. Results and Discussion

### 2.1. Disc Diffusion Method

The MSKE displayed antimicrobial activity against both *S. aureus* ATCC 25923 and all of the 19 tested MRSA strains as shown by the presence of inhibition zones in the disc diffusion study in Table 1.

**Table 1:** Antibacterial activity of MSKE against *S. aureus* ATCC 25923 and 19 clinical MRSA isolates (nz = no inhibition zone,^a^ mean values ± S.D. of triplicate results, ^b^ mean values ± S.D. from 19 MRSA strains,*no significant difference from *S. aureus* ATCC 25923 at *p* > 0.01).

Bacterial strains	Mean diameter of inhibition zone (mm)^a^					
	Control (solvents)	MSKE				Vancomycin 30 µg/disc
		0.625 mg/disc	1.25 mg/disc	2.50 mg/disc	5.00 mg/disc	
***S. aureus*** **ATCC 25923**	nz	11.44 ± 0.59	14.81 ± 0.49	13.94 ± 1.34	17.06 ± 3.23	18.31 ± 0.52
**Clinical MRSA strains**						
M 01	nz	12.00 ± 0.14	14.08 ± 0.25	14.17 ± 0.23	18.25 ± 1.14	21.00 ± 0.33
M 02	nz	12.03 ± 0.19	14.11 ± 0.46	17.20 ± 2.31	17.92 ± 1.53	20.44 ± 2.50
M 03	nz	11.92 ± 0.68	15.58 ± 0.51	15.53 ± 0.90	20.50 ± 2.28	22.89 ± 2.01
M 04	nz	11.28 ± 0.38	14.08 ± 0.82	14.56 ± 0.55	18.19 ± 1.61	19.67 ± 0.33
M 05	nz	11.58 ± 0.52	13.69 ± 0.35	13.20 ± 0.52	16.03 ± 0.20	19.78 ± 0.19
M 06	nz	11.92 ± 0.82	14.89 ± 0.71	14.50 ± 1.65	18.78 ± 2.67	19.33 ± 0.67
M 07	nz	10.92 ± 0.46	14.28 ± 0.57	14.33 ± 0.71	18.53 ± 0.91	20.89 ± 0.84
M 08	nz	11.31 ± 0.51	14.36 ± 0.27	15.59 ± 0.52	19.34 ± 0.54	19.78 ± 0.69
M 09	nz	10.39 ± 0.46	13.08 ± 0.76	13.36 ± 0.51	17.97 ± 0.20	20.44 ± 1.95
M 10	nz	12.44 ± 0.51	15.72 ± 1.50	14.52 ± 0.35	18.36 ± 0.20	18.33 ± 0.58
M 11	nz	8.17 ± 0.29	10.06 ± 0.10	12.17 ± 0.76	13.56 ± 0.51	18.61 ± 0.54
M 12	nz	10.33 ± 0.44	12.11 ± 0.10	14.11 ± 0.25	15.72 ± 0.10	19.11 ± 1.07
M 13	nz	9.11 ± 0.10	10.72 ± 0.59	12.72 ± 0.69	14.72 ± 0.86	18.33 ± 1.04
M 14	nz	8.56 ± 0.35	10.56 ± 0.35	13.00 ± 0.17	14.61 ± 0.54	21.89 ± 1.95
M 15	nz	10.44 ± 0.67	12.39 ± 0.54	14.22 ± 1.51	16.83 ± 1.09	20.61 ± 1.69
M 16	nz	9.56 ± 0.25	11.00 ± 0.29	13.67 ± 0.33	15.39 ± 0.38	19.67 ± 0.73
M 17	nz	10.28 ± 0.19	11.61 ± 0.42	13.61 ± 0.69	15.39 ± 0.54	19.28 ± 0.54
M 18	nz	9.44 ± 1.08	10.83 ± 0.60	12.61 ± 0.92	15.00 ± 0.76	20.44 ± 1.80
M 19	nz	9.94 ± 1.55	11.28 ± 1.44	12.89 ± 1.55	15.06 ± 1.40	18.72 ± 0.92
**Mean MRSA (n = 19) ^b^**	nz	10.61 ± 1.25*	12.87 ±1.82*	14.00 ±1.21*	16.85 ±1.94*	19.96 ± 1.20*

The mean diameter of the inhibition zone was increased from 11.44 ± 0.59 to 14.81 ± 0.49, 13.94 ± 1.34 and 17.06 ± 3.23 mm for *S. aureus* ATCC 25923 over an increase in concentration of 0.625 to 1.25, 2.50 and 5.00 mg/disc, respectively. The uneven increase trend in the inhibition zones could be a result of uneven drug diffusion from the paper disc to agar plate. 

At the same concentration range, similar mean inhibition zones (10.61 ± 1.25 to 12.87 ± 1.82, 14.00 ± 1.21 and 16.85 ± 1.94 mm) were found for all 19 clinical MRSA isolates. There was no significant difference between the mean inhibition zone of the reference MSSA strain and the MRSA strains (*p* > 0.01). The solvent used, 10% DMSO, produced no visible inhibition zone in this study, whereas the positive control, vancomycin at 30 μg/disc, produced mean inhibition zones of 18.31 ± 0.52 mm for the standard MSSA strain and 19.96 ± 1.20 mm for the 19 MRSA strains.

### 2.2. The Minimum Inhibitory Concentrations (MICs) and Minimum Bactericidal Concentrations (MBCs)

Table 2 shows the MIC and MBC values of MSKE and its phenolic principles (PGG, GA, MG) against *S. aureus* ATCC 25923 and 19 clinical MRSA isolates as compared to the reference antibiotics. The MIC and the MBC values of these phenolic principles against the standard MSSA strain were lower than those generated by MSKE. 

The order of potency for MICs was PGG (0.13 ± 0.00 mg/mL) < GA (0.19 ± 0.00 mg/mL) < MG (0.38 ± 0.00 mg/mL) < MSKE (0.47 ± 0.00 mg/mL). The order of potency for MBCs was PGG (0.50 ± 0.00 mg/mL) < GA (0.75 ± 0.00 mg/mL) < MG (1.00 ± 0.00 mg/mL) < MSKE (1.88 ± 0.00 mg/mL). For the 19 clinical MRSA isolates, the MICs of MSKE and its phenolic principles against these MRSA strains ranged from 0.13 ± 0.00 to 0.25 ± 0.00 mg/mL for PGG, 0.47 ± 0.00 mg/mL for MSKE, 0.19 ± 0.00 to >3.00 ± 0.00 mg/mL for GA, and 0.75 ± 0.00 to 2.50 ± 0.87 mg/mL for MG. 

The MBC values were as follows: PGG 1.00 ± 0.00 to >1.00 ± 0.00 mg/mL, MG 1.00 ± 0.43 to 2.50 ± 0.87 mg/mL, GA 0.38 ± 0.00 to >3.00 ± 0.00 mg/mL and MSKE 0.94 ± 0.00 to 3.75 ± 0.00 mg/mL. For the reference antibiotics, vancomycin exhibited both lower MIC (0.78 ± 0.00 µg/mL) and MBC (3.13 ± 0.00 µg/mL) values than penicillin G (MIC and MBC = 41.67 ± 18.04 µg/mL) against the standard MSSA strain. All 19 MRSA isolates were resistant to penicillin G with MIC and MBC values ranging from 125.00 ± 0.00 to 8,000.00 ± 0.00 µg/mL. The 19 MRSA isolates were susceptible to vancomycin with MIC and MBC values ranging from 1.56 ± 0.00 to 6.25 ± 0.00 µg/mL and 3.13 ± 0.00 to 12.50 ± 0.00 µg/mL, respectively.

These broth microdilution results agreed with the disc diffusion assays, which demonstrated that the antibacterial activity of MSKE against the standard MSSA strain and all of the clinical MRSA strains were not significantly different (*p* > 0.01) as shown by their similar MBC mean values (1.88 ± 0.00 and 1.83 ± 0.79 mg/mL, respectively) and equivalent MIC value (0.47 ± 0.00 mg/mL) (Table 2). Among the phenolic principles of MSKE, PGG exhibited the most potent antibacterial activity against both the standard MSSA strain and all 19 clinically isolated strains, exhibiting the lowest mean MIC values (0.13 ± 0.00 and 0.16 ± 0.03 mg/mL, respectively) compared to GA (0.19 ± 0.00 and >1.07 ± 1.19 mg/mL, respectively) and MG (0.38 ± 0.00 and 1.33 ± 0.34 mg/mL, respectively). 

We observed that the mean MIC of PGG against all tested strains was approximately 3−fold lower than that of MSKE. Similarly, PGG also exhibited lower mean MBC values (>1.00 ± 0.00 mg/mL) against all 19 MRSA strains compared to GA (>1.25 ± 1.09 mg/mL) and MG (1.42 ± 0.32 mg/mL). Because PGG is the major principle of MSKE, present at the highest concentration at approximately 65% and GA and MG are present at only trace levels (0.88 and 0.62%, respectively), the data imply that PGG may be the major contributor to the antibacterial activity of MSKE; there may also be other unidentified constituents of MSKE that possess lower antibacterial potencies than PGG that have not yet been isolated. 

**Table 2:** The mean MICs and MBCs of MSKE and its phenolic principles against *S. aureus* ATCC 25923 and 19 clinically isolated MRSA strains (^a^ mean values ± S.D. of triplicate results, ^b^ mean values ± S.D. from 19 MRSA strains).

Bacterial strains	Susceptibility of Bacteria^a^											
	MSKE (mg/mL)		PGG (mg/mL)		MG (mg/mL)		GA (mg/mL)		Vancomycin (µg/mL)		Penicillin G (µg/mL)	
	MIC	MBC	MIC	MBC	MIC	MBC	MIC	MBC	MIC	MBC	MIC	MBC
***S. aureus*** ** ATCC 25923**	0.47 ± 0.00	1.88 ± 0.00	0.13 ± 0.00	0.50 ± 0.00	0.38 ± 0.00	1.00 ± 0.00	0.19 ± 0.00	0.75 ± 0.00	0.78 ± 0.00	3.13 ± 0.00	41.67 ± 18.04	41.67 ± 18.04
**Clinical MRSA strains**												
M 01	0.47 ± 0.00	1.88 ± 0.00	0.17 ± 0.07	1.00 ± 0.00	1.25 ± 0.43	1.50 ± 0.00	0.25 ± 0.11	0.75 ± 0.00	1.56 ± 0.00	6.25 ± 0.00	2,000.00 ± 0.00	8,000.00 ± 0.00
M 02	0.47 ± 0.00	1.88 ± 0.00	0.17 ± 0.07	1.00 ± 0.00	1.00 ± 0.43	1.25 ± 0.43	0.38 ± 0.00	0.63 ± 0.22	1.56 ± 0.00	6.25 ± 0.00	2,000.00 ± 0.00	5,333.33 ± 2,309.40
M 03	0.47 ± 0.00	1.88 ± 0.00	0.13 ± 0.00	1.00 ± 0.00	1.25 ± 0.43	1.25 ± 0.43	0.38 ± 0.00	0.63 ± 0.22	1.56 ± 0.00	6.25 ± 0.00	2,000.00 ± 0.00	4,000.00 ± 0.00
M 04	0.47 ± 0.00	1.88 ± 0.00	0.13 ± 0.00	1.00 ± 0.00	1.25 ± 0.43	1.25 ± 0.43	0.25 ± 0.11	0.50 ± 0.22	1.56 ± 0.00	6.25 ± 0.00	1,333.33 ± 577.35	2,666.67 ± 1,154.70
M 05	0.47 ± 0.00	1.88 ± 0.00	0.13 ± 0.00	1.00 ± 0.00	1.25 ± 0.43	1.50 ± 0.00	0.38 ± 0.00	1.00 ± 0.43	1.56 ± 0.00	6.25 ± 0.00	2,000.00 ± 0.00	5,333.33 ± 2,309.40
M 06	0.47 ± 0.00	1.88 ± 0.00	0.17 ± 0.07	1.00 ± 0.00	1.25 ± 0.43	1.25 ± 0.43	0.25 ± 0.11	0.38 ± 0.00	1.56 ± 0.00	12.50 ± 0.00	1,666.67 ± 577.35	4,000.00 ± 0.00
M 07	0.47 ± 0.00	1.88 ± 0.00	0.17 ± 0.07	1.00 ± 0.00	1.25 ± 0.43	1.75 ± 1.15	0.63 ± 0.22	0.75 ± 0.00	1.56 ± 0.00	6.25 ± 0.00	2,000.00 ± 0.00	8,000.00 ± 0.00
M 08	0.47 ± 0.00	1.88 ± 0.00	0.17 ± 0.07	>1.00 ± 0.00	1.25 ± 0.43	1.25 ± 0.43	0.38 ± 0.00	0.38 ± 0.00	1.56 ± 0.00	6.25 ± 0.00	1,666.67 ± 577.35	4,000.00 ± 0.00
M 09	0.47 ± 0.00	1.88 ± 0.00	0.17 ± 0.07	1.00 ± 0.00	1.25 ± 0.43	1.25 ± 0.43	0.38 ± 0.00	0.38 ± 0.00	1.56 ± 0.00	12.50 ± 0.00	1,666.67 ± 577.35	4,000.00 ± 0.00
M 10	0.47 ± 0.00	1.88 ± 0.00	0.17 ± 0.07	>1.00 ± 0.00	1.25 ± 0.43	1.50 ± 0.00	0.63 ± 0.22	0.75 ± 0.00	1.56 ± 0.00	6.25 ± 0.00	1,666.67 ± 577.35	8,000.00 ± 0.00
M 11	0.47 ± 0.00	1.88 ± 0.00	0.17 ± 0.07	1.00 ± 0.00	1.50 ± 0.00	1.50 ± 0.00	>3.00 ± 0.00	>3.00 ± 0.00	1.56 ± 0.00	12.50 ± 0.00	2,000.00 ± 0.00	8,000.00 ± 0.00
M 12	0.47 ± 0.00	3.75 ± 0.00	0.13 ± 0.00	>1.00 ± 0.00	2.50 ± 0.87	2.50 ± 0.87	>3.00 ± 0.00	>3.00 ± 0.00	1.56 ± 0.00	12.50 ± 0.00	4,000.00 ± 0.00	8,000.00 ± 0.00
M 13	0.47 ± 0.00	3.75 ± 0.00	0.17 ± 0.07	1.00 ± 0.00	0.75 ± 0.00	1.00 ± 0.43	0.25 ± 0.11	0.75 ± 0.00	6.25 ± 0.00	12.50 ± 0.00	8,000.00 ± 0.00	8,000.00 ± 0.00
M 14	0.47 ± 0.00	0.94 ± 0.00	0.13 ± 0.00	1.00 ± 0.00	1.25 ± 0.43	1.25 ± 0.43	0.63 ± 0.22	0.75 ± 0.00	3.13 ± 0.00	12.50 ± 0.00	4,000.00 ± 0.00	8,000.00 ± 0.00
M 15	0.47 ± 0.00	0.94 ± 0.00	0.13 ± 0.00	1.00 ± 0.00	1.50 ± 0.00	1.50 ± 0.00	>3.00 ± 0.00	>3.00 ± 0.00	3.13 ± 0.00	12.50 ± 0.00	125.00 ± 0.00	125.00 ± 0.00
M 16	0.47 ± 0.00	0.94 ± 0.00	0.25 ± 0.00	1.00 ± 0.00	1.50 ± 0.00	1.50 ± 0.00	>3.00 ± 0.00	>3.00 ± 0.00	3.13 ± 0.00	12.50 ± 0.00	4,000.00 ± 0.00	8,000.00 ± 0.00
M 17	0.47 ± 0.00	0.94 ± 0.00	0.13 ± 0.00	1.00 ± 0.00	1.00 ± 0.43	1.00 ± 0.43	0.31 ± 0.11	0.75 ± 0.00	3.13 ± 0.00	12.50 ± 0.00	125.00 ± 0.00	125.00 ± 0.00
M 18	0.47 ± 0.00	0.94 ± 0.00	0.17 ± 0.07	1.00 ± 0.00	1.50 ± 0.00	1.50 ± 0.00	0.19 ± 0.00	0.38 ± 0.00	3.13 ± 0.00	12.50 ± 0.00	2,000.00± 0.00	8,000.00 ± 0.00
M 19	0.47 ± 0.00	1.88 ± 0.00	0.17 ± 0.07	1.00 ± 0.00	1.50 ± 0.00	1.50 ± 0.00	>3.00 ± 0.00	>3.00 ± 0.00	1.56 ± 0.00	3.13 ± 0.00	2,000.00± 0.00	8,000.00 ± 0.00
**Mean MRSA (n = 19) ^b^**	0.47 ± 0.00	1.83 ± 0.79	0.16 ± 0.03	>1.00 ± 0.00	1.33 ± 0.34	1.42 ± 0.32	>1.07 ± 1.19	>1.25 ± 1.09	2.22 ± 1.20	9.38 ± 3.45	2,328.95 ± 1,729.87	5,767.54 ± 2,748.78

### 2.3. Time–Kill Curves

**Figure 1:**
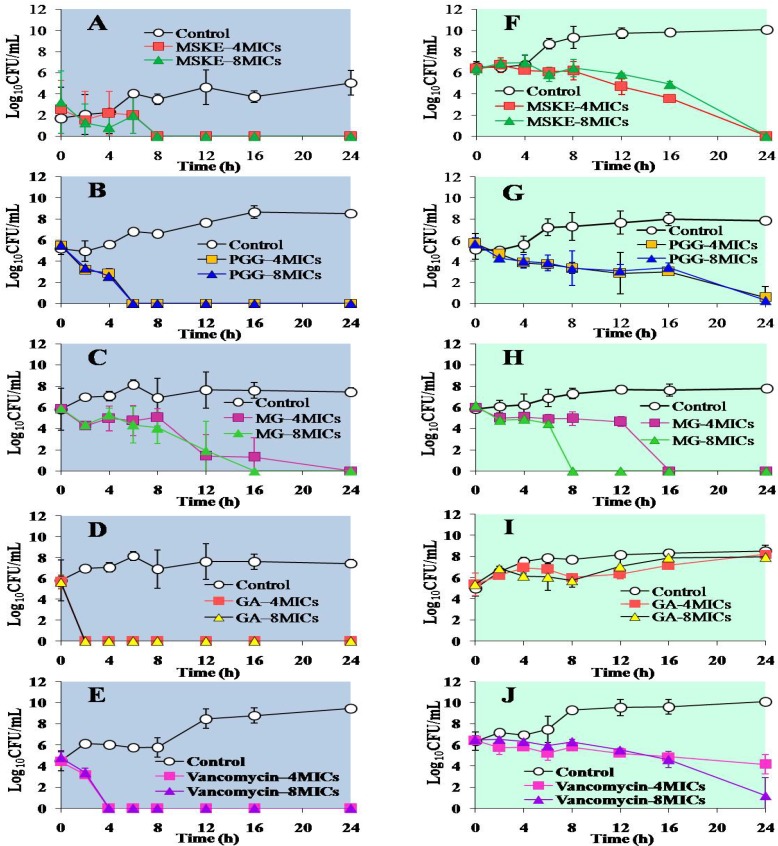
Time–kill curves of *S. aureus* ATCC 25923 (**A–E**) and the M03 MRSA strain (**F–J**) after treatment with MSKE (**A and F**), PGG (**B and G**), MG (**C and H**), GA (**D and I**) and vancomycin (**E and J**). Each symbol indicates the mean ± S.D. for at least duplicate samples.

The bactericidal effects of MSKE and its phenolic principles against standard MSSA strain are shown in Figure 1A–Figure 1E. The time−kill curves of MSKE and its principles against the M03 MRSA strain are shown in Figure 1F− Figure 1J.

Using concentrations of 4 and 8 MICs; we showed that for the treatment of standard MSSA (Figure 1A–Figure 1E), MSKE, PGG and GA induced complete cell death within 8, 6 and 2 h, respectively, whereas vancomycin exhibited complete eradication within 4 h. MG was able to kill MSSA cells completely within 24 and 16 h at concentrations of 4 and 8 MICs, respectively. Treatment of M03 MRSA (Figure 1F–Figure 1J) with either MSKE or PGG at 4 and 8 MICs exerted the most bactericidal activity (≥5 log_10_–fold decrease) within 24 h, whereas treatment with MG using the same concentration range resulted in complete cell death within 16 and 8 h, respectively. GA produced no bactericidal effects on the M03 MRSA strain at any time and exerted only a 1–2 log_10_ reduction within 8 h compared with controls (Figure 1I). In the case of vancomycin, a bacteriostatic effect on M03 MRSA was detected by 24 h after incubation at 4 MICs, whereas a bactericidal effect was observed at 8 MICs within 24 h.

The ability of MSKE, PGG and GA to kill the standard MSSA strain at 4 and 8 MICs occurred in a time–dependent manner, whereas the bactericidal activity of MG demonstrated a concentration− and time−dependent pattern. Although the killing rate of GA against the standard MSSA strain at 4 and 8 MICs was time−dependent, GA failed to inhibit the growth of the M03 MRSA strain at either concentration, which is not in agreement with the MBC values. This finding implied that the MIC and MBC values resulting from the broth microdilution study that provided a static view may disagree with the results from the time–kill assays that measured the killing rates of the microorganism in a dynamic manner [9]. This phenomenon may also be explained by the survival of adaptive, resistant forms such as those that have been observed after incubating *Enterococcus faecalis* with the chloroform fraction of rhizomes from *Aristolochia paucinervis* Pomel [10].

### 2.4. Electron Microscopy

**Figure 2:**
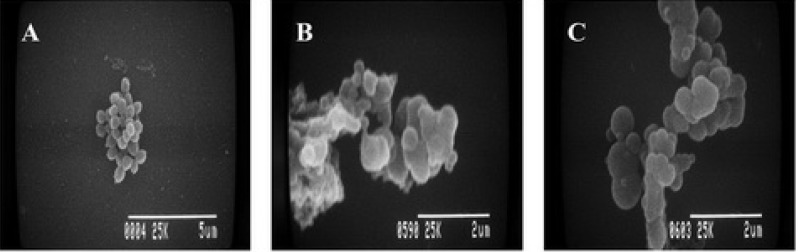
Scanning electron micrographs of *S. aureus* ATCC 25923 at 12 h after treatment with (**A**) 1% DMSO (control), (**B**) MSKE at 4 MICs and (**C**) PGG at 4 MICs.

Scanning electron microscopy (SEM) images of the standard MSSA strain are shown in Figure 2. The SEM images revealed that MSKE and PGG induced an alteration in cell morphology. Control cells in the presence of 1% DMSO showed a spherical shape in grapelike clusters (Figure 2A). In contrast, cells treated with either MSKE or PGG at a MIC of 4 displayed clusters of non–separated cells (Figure 2B and Figure 2C). 

**Figure 3:**
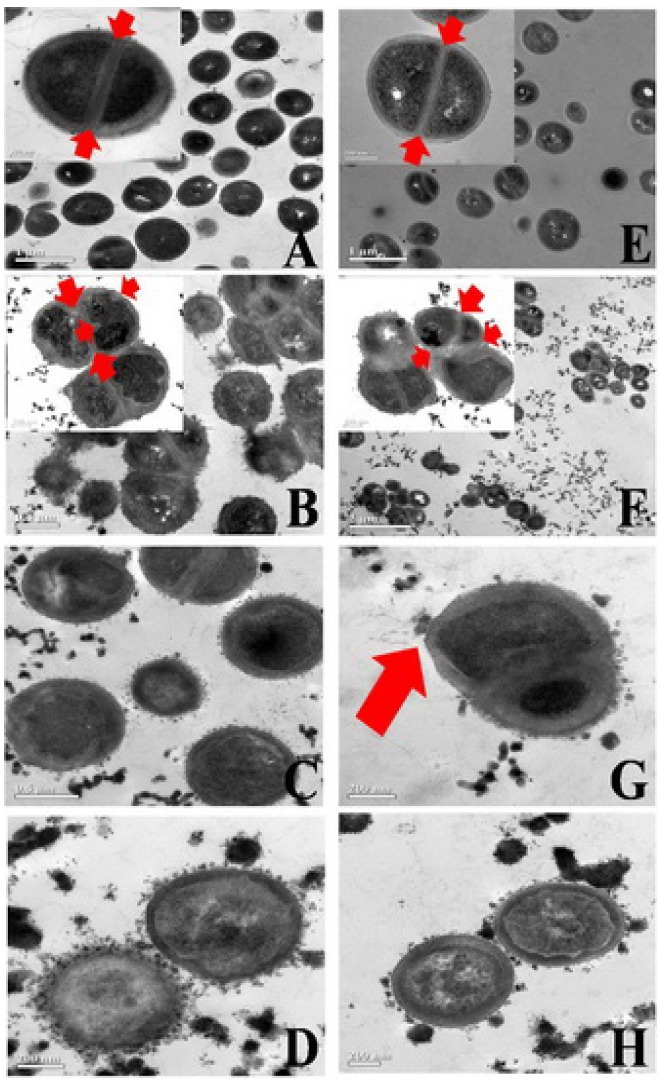
Transmission electron micrographs of *S. aureus* ATCC 25923 (**A–D**) and the M09 MRSA strain (**E–H**) 12 h after treatment with different concentrations of MSKE, 1 MIC (**B, F**) 2 MICs (**C, G**) and 4 MICs (**D, H**), when compared to the control, 1% DMSO, (**A, E**).

The SEM images indicated an impaired cell division of the tested microorganisms after treatment with MSKE and were in agreement with the results obtained from transmission electron microscopy (TEM) images, which demonstrated ultra–structural changes in the tested MSSA cells (Figure 3B–Figure 3D) and M09 MRSA cells (Figure 3F–Figure 3H) after treatment with MSKE. At the MIC of MSKE, the separation of daughter cells was severely inhibited as demonstrated by the incomplete septum formation; this phenomenon is demonstrated by the pseudomulticellular appearance and asymmetrical initiation of septum formation that are visible in groups of non–separated cells (Figure 3B and Figure 3F, arrows). 

In addition, the thickness of the bacterial cell walls was significantly increased following treatment. These damaging effects and the observed ultra–structural changes appeared to be more prominent with the increase in MSKE concentration from 1 to 2 or 4 MICs. The splitting of cell materials to the outer surface of the disrupted membrane (Figure 3C, Figure 3D, Figure 3G and Figure 3H) and the fibrous matrix extending from the surface of the treated cells (Figure 3G, arrow) are obviously seen. The untreated cells of both standard MSSA and M09 MRSA strains showed symmetrical initiation and completed septum formation (Figure 3A and Figure 3E, arrows). These results were similar to the ultra–structural changes observed in the MRSA strains treated with *Quercus infectoria* extract, tannic acid [11] or green tea extract (*Camellia sinensis*) [12]. Since polyphenols are known to form complexes with proteins and polysaccharides [13], bacterial surfaces have the ability to bind large amounts of polyphenols [14,15]. The inhibitory mechanism of MSKE and its phenolic principles against MSSA and MRSA strains may therefore be due to the damage to the bacterial membrane. This leads to permeability of the outer and inner membranes of treated cells and disruption of membranes, resulting in the release of small cellular molecules. This hypothesis is in accordance with the inhibitory mechanism of tea polyphenols towards *Pseudomonas aeruginosa* suggested by Yi *et al.* [16].

### 2.5. The Enhancing Effects of MSKE and Its Phenolic Principles on the Antibacterial Activity of Penicillin G

Table 3 demonstrates the capabilities of MSKE and its phenolic principles to enhance the antibacterial activity of penicillin G. It was found that the addition of MSKE at 0.25 or 0.5 MIC to penicillin G led to a marked reduction in the MIC of penicillin G against the standard MSSA strain from 41.67 ± 18.04 to 3.91 ± 0.00 and 1.95 ± 0.00 µg/mL, respectively. The MIC of penicillin G was also reduced when it was combined with 0.25 and 0.5 MICs of the phenolic principles, showing reductions from 41.67 ± 18.04 to 7.81 ± 0.00 µg/mL at both concentrations for PGG, to 15.63 ± 0.00 and 7.81 ± 0.00 µg/mL, respectively, for GA and to 31.25 ± 0.00 and 15.63 ± 0.00, respectively, for MG. A similar enhancement effect was also observed in the antibacterial activity of penicillin G against 19 clinical MRSA strains when it was combined with 0.25 and 0.5 MICs of MSKE and its phenolic principles. The mean MIC of penicillin G against the 19 MRSA strains at the two concentrations was reduced at least 5–fold from 2,328.95 ± 1,729.87 µg/mL to 361.84 ± 159.45 and 309.21 ± 145.05 µg/mL, respectively, for MSKE, to 409.95 ± 300.04 and 324.42 ± 222.60 µg/mL, respectively, for PGG, to 250.41 ± 439.73 and 9.16 ± 28.08 µg/mL, respectively, for MG and to 244.04 ± 205.36 and 237.46 ± 207.16 µg/mL, respectively, for GA. It was noted that the addition of MG at 0.5 MIC dramatically reduced the mean MIC of penicillin G against the 19 clinical MRSA strains of approximately 254−fold from 2,328.95 ± 1,729.87 to 9.16 ± 28.08 µg/mL. These results suggest that despite the weak antimicrobial effects of MG against MRSA and MSSA, MG may have the capacity to enhance the bacterial susceptibility to penicillin G similarly to the alkyl gallates, which have been shown to dramatically intensify susceptibility to β–lactams in MSSA and MRSA strains [17].

These results imply that the enhancing ability of MSKE on the antibacterial activity of penicillin G against MRSA strains may be due to the major phenolic principle, PGG, as the capacity of PGG to reduce the mean MIC of penicillin G against MRSA strains was close to the capacity of MSKE at both 0.25 and 0.5 MICs. Although MG and GA alone exhibited lower antibacterial activity than PGG, GA and MG at 0.25 and 0.5 MICs in combination with penicillin G demonstrated higher capacities to reduce the mean MIC of penicillin G against MRSA strains than the addition of PGG at the same concentration (Table 3). This finding suggests that the enhancing capacity of MSKE and its phenolic principles may not be related only to their antibacterial activity.

Moreover, the combination of 0.5 or 1 MIC of MSKE with penicillin G did not enhance the bactericidal activity of penicillin G against the 19 clinical MRSA isolates. Their mean MBCs were 5,767.54 ± 2,748.78 for penicillin G alone, 5,907.89 ± 2,757.52 when 0.5 MIC of MSKE was added and 5,907.89 ± 2,757.52 µg/mL when 1 MIC of MSKE was added (Table 4). For the standard MSSA strain, the mean MBC of penicillin G was reduced from 41.67 ± 18.04 to 31.25 ± 0.00 µg/mL with the addition of either 0.5 or 1 MIC of MSKE. This result implies that MSKE can enhance the antibacterial activity of penicillin G against MRSA with a bacteriostatic effect but not a bactericidal effect. However, the enhancing mechanism of MSKE and its phenolic principles on the antibacterial activity of penicillin G against MRSA and MSSA has not been elucidated, but it could be similar to tellimagrandin I and corilagin, which enhanced the antibacterial activity of β−lactam antibiotics by inactivating the penicillin−binding protein and suppressing the activity of β−lactamase [18].

**Table 3:** The enhancing effects of MSKE and its phenolic principles on the antibacterial activities of penicillin G against *S. aureus* ATCC 25923 and 19 clinical MRSA isolates (^a^ mean values ± S.D. of triplicate results, ^b^ mean values ± S.D.from 19 MRSA strains).

Bacterial strains	MIC of Penicillin G (µg/mL)^a^								
	Penicillin G alone	+MSKE 0.25MIC	+MSKE 0.5MIC	+PGG 0.25MIC	+PGG 0.5MIC	+MG 0.25MIC	+MG 0.5MIC	+GA 0.25MIC	+GA 0.5MIC
***S. aureus* ATCC 25923**	41.67 ± 18.04	3.91 ± 0.00	1.95 ± 0.00	7.81 ± 0.00	7.81 ± 0.00	31.25 ± 0.00	15.63 ± 0.00	15.63 ± 0.00	7.81 ± 0.00
**Clinical MRSA strains**									
M 01	2,000.00 ± 0.00	500.00 ± 0.00	250.00 ± 0.00	250.00 ± 0.00	250.00 ± 0.00	62.50 ± 0.00	1.95 ± 0.00	250.00 ± 0.00	250.00 ± 0.00
M 02	2,000.00 ± 0.00	250.00 ± 0.00	250.00 ± 0.00	250.00 ± 0.00	250.00 ± 0.00	125.00 ± 0.00	1.95 ± 0.00	250.00 ± 0.00	125.00 ± 0.00
M 03	2,000.00 ± 0.00	500.00 ± 0.00	250.00 ± 0.00	1,000.00 ± 0.00	500.00 ± 0.00	125.00 ± 0.00	1.95 ± 0.00	250.00 ± 0.00	250.00 ± 0.00
M 04	1,333.33 ± 577.35	250.00 ± 0.00	250.00 ± 0.00	250.00 ± 0.00	250.00 ± 0.00	125.00 ± 0.00	1.95 ± 0.00	250.00 ± 0.00	250.00 ± 0.00
M 05	2,000.00 ± 0.00	250.00 ± 0.00	250.00 ± 0.00	250.00 ± 0.00	250.00 ± 0.00	125.00 ± 0.00	1.95 ± 0.00	250.00 ± 0.00	250.00 ± 0.00
M 06	1,666.67 ± 577.35	250.00 ± 0.00	250.00 ± 0.00	500.00 ± 0.00	250.00 ± 0.00	62.50 ± 0.00	1.95 ± 0.00	3.91 ± 0.00	3.91 ± 0.00
M 07	2,000.00 ± 0.00	500.00 ± 0.00	250.00 ± 0.00	250.00 ± 0.00	250.00 ± 0.00	125.00 ± 0.00	1.95 ± 0.00	250.00 ± 0.00	250.00 ± 0.00
M 08	1,666.67 ± 577.35	250.00 ± 0.00	250.00 ± 0.00	1,000.00 ± 0.00	500.00 ± 0.00	250.00 ± 0.00	125.00 ± 0.00	250.00 ± 0.00	250.00 ± 0.00
M 09	1,666.67 ± 577.35	250.00 ± 0.00	250.00 ± 0.00	250.00 ± 0.00	250.00 ± 0.00	250.00 ± 0.00	3.91 ± 0.00	250.00 ± 0.00	250.00 ± 0.00
M 10	1,666.67 ± 577.35	250.00 ± 0.00	250.00 ± 0.00	250.00 ± 0.00	125.00 ± 0.00	125.00 ± 0.00	3.91 ± 0.00	250.00 ± 0.00	250.00 ± 0.00
M 11	2,000.00 ± 0.00	500.00 ± 0.00	500.00 ± 0.00	500.00 ± 0.00	250.00 ± 0.00	250.00 ± 0.00	3.91 ± 0.00	250.00 ± 0.00	250.00 ± 0.00
M 12	4,000.00 ± 0.00	500.00 ± 0.00	500.00 ± 0.00	1,000.00 ± 0.00	1,000.00 ± 0.00	2,000.00 ± 0.00	3.91 ± 0.00	1,000.00 ± 0.00	1,000.00 ± 0.00
M 13	8,000.00 ± 0.00	500.00 ± 0.00	500.00 ± 0.00	250.00 ± 0.00	250.00 ± 0.00	250.00 ± 0.00	3.91 ± 0.00	125.00 ± 0.00	125.00 ± 0.00
M 14	4,000.00 ± 0.00	500.00 ± 0.00	500.00 ± 0.00	500.00 ± 0.00	500.00 ± 0.00	250.00 ± 0.00	3.91 ± 0.00	250.00 ± 0.00	250.00 ± 0.00
M 15	125.00 ± 0.00	62.50 ± 0.00	62.50 ± 0.00	7.80 ± 0.00	7.80 ± 0.00	3.91 ± 0.00	0.06 ± 0.00	3.91 ± 0.00	3.91 ± 0.00
M 16	4,000.00 ± 0.00	500.00 ± 0.00	500.00 ± 0.00	500.00 ± 0.00	500.00 ± 0.00	500.00 ± 0.00	3.91 ± 0.00	250.00 ± 0.00	250.00 ± 0.00
M 17	125.00 ± 0.00	62.50 ± 0.00	62.50 ± 0.00	31.25 ± 0.00	31.25 ± 0.00	3.91 ± 0.00	0.06 ± 0.00	3.91 ± 0.00	3.91 ± 0.00
M 18	2,000.00 ± 0.00	500.00 ± 0.00	250.00 ± 0.00	500.00 ± 0.00	500.00 ± 0.00	62.50 ± 0.00	3.91 ± 0.00	250.00 ± 0.00	250.00 ± 0.00
M 19	2,000.00 ± 0.00	500.00 ± 0.00	500.00 ± 0.00	250.00 ± 0.00	250.00 ± 0.00	62.50 ± 0.00	3.91 ± 0.00	250.00 ± 0.00	250.00 ± 0.00
**Mean MRSA (n = 19) ^b^**	2,328.95 ± 1,729.87	361.84 ± 159.45	309.21 ±145.05	409.95 ± 300.04	324.42 ± 222.60	250.41 ± 439.73	9.16 ± 28.08	244.04 ± 205.36	237.46 ± 207.16

**Table 4:** Effects of MSKE and its phenolic principles on the MBCs of penicillin G against *S. aureus* ATCC 25923 and clinical MRSA strains (^a^ mean values ± S.D. of triplicate results, ^b^ mean values ± S.D. from 19 MRSA strains).

Bacterial strains	MBC of Penicillin G (µg/mL) ^a^		
	Penicillin G alone	with MSKE 0.5 MIC	with MSKE 1 MIC
***S. aureus* ATCC 25923**	41.67 ± 18.04	31.25 ± 0.00	31.25 ± 0.00
**Clinical MRSA strains**			
M 01	8,000.00 ± 0.00	8,000.00 ± 0.00	8,000.00 ± 0.00
M 02	5,333.33 ± 2,309.40	4,000.00 ± 0.00	4,000.00 ± 0.00
M 03	4,000.00 ± 0.00	4,000.00 ± 0.00	4,000.00 ± 0.00
M 04	2,666.67 ± 1,154.70	4,000.00 ± 0.00	4,000.00 ± 0.00
M 05	5,333.33 ± 2,309.40	8,000.00 ± 0.00	8,000.00 ± 0.00
M 06	4,000.00 ± 0.00	4,000.00 ± 0.00	4,000.00 ± 0.00
M 07	8,000.00 ± 0.00	8,000.00 ± 0.00	8,000.00 ± 0.00
M 08	4,000.00 ± 0.00	4,000.00 ± 0.00	4,000.00 ± 0.00
M 09	4,000.00 ± 0.00	4,000.00 ± 0.00	4,000.00 ± 0.00
M 10	8,000.00 ± 0.00	8,000.00 ± 0.00	8,000.00 ± 0.00
M 11	8,000.00 ± 0.00	8,000.00 ± 0.00	8,000.00 ± 0.00
M 12	8,000.00 ± 0.00	8,000.00 ± 0.00	8,000.00 ± 0.00
M 13	8,000.00 ± 0.00	8,000.00 ± 0.00	8,000.00 ± 0.00
M 14	8,000.00 ± 0.00	8,000.00 ± 0.00	8,000.00 ± 0.00
M 15	125.00 ± 0.00	125.00 ± 0.00	125.00 ± 0.00
M 16	8,000.00 ± 0.00	8,000.00 ± 0.00	8,000.00 ± 0.00
M 17	125.00 ± 0.00	125.00 ± 0.00	125.00 ± 0.00
M 18	8,000.00 ± 0.00	8,000.00 ± 0.00	8,000.00 ± 0.00
M 19	8,000.00 ± 0.00	8,000.00 ± 0.00	8,000.00 ± 0.00
**Mean MRSA (n = 19) ^b^**	5,767.54 ± 2,748.78	5,907.89 ± 2,757.52	5,907.89 ± 2,757.52

## 3. Experimental

### 3.1. Materials

#### 3.1.1. Test Materials

Gallic acid (GA; ≥98% purity) and methyl gallate (MG; ≥98% purity) were purchased from Fluka (Buchs, Switzerland). Pentagalloylglucopyranose (PGG; >95% purity) was purchased from Endotherm BmbH (Germany). Penicillin G (1651 U/mg) was USP grade and was purchased from Bio Basic Inc. (Canada). Vancomycin for injection (1,055 µg/mg) was obtained from CJ Cheiljedang Corp (Kyunggi−Do, Korea). Reference discs of vancomycin were produced by Oxoid (Basingstoke, UK). Other chemicals and solvents were of analytical grade and obtained from local distributors.

#### 3.1.2. Plant Material and Preparation of Plant Extract

Fully grown, unripened Thai mango fruits (*Mangifera indica* L. cv. ‘Fahlun’, Anacardiaceae) were purchased from a local market. The seed kernels were removed and extracted following the method of Nithitanakool *et al.* [5]. Briefly, the kernels were homogenised in hot ethanol (80 °C) and defatted with hexane. After the solvents were evaporated, the remaining aqueous residue was freeze–dried to generate a crude mango seed kernel extract (MSKE) with a yield of 6.69% w/w (on the basis of wet weight). MSKE and its isolated compounds were dissolved in 10% aqueous dimethyl sulfoxide (DMSO) before use.

#### 3.1.3. Standardisation

The MSKE was standardised with regard to the content of its three polyphenolic compounds (PGG, GA and MG) using the thin layer chromatographic (TLC)−UV densitometric method [5]. Briefly, an aliquot of MSKE (25 mg/mL) was applied to TLC plates together with serial dilutions of the standard solutions of PGG, GA and MG. The TLC plates were then developed in a pre-saturated TLC tank containing CHCl_3_/ethanol/formic acid (3:5:1, v/v/v) as the mobile phase for PGG and CHCl_3_/methanol/ethyl acetate/ethyl methyl ketone/formic acid (6:1.6:2:2:5, v/v/v/v/drop) for GA and MG. The developed TLC plates were scanned using a TLC densitometer at 286 nm, and the amount of each compound in the MSKE (PGG 65.61 ± 0.95%, GA 0.88 ± 0.16% and MG 0.62 ± 0.09% w/w based on dry weight) was calculated from the calibration curves.

#### 3.1.4. Bacterial Strains Tested

A total of 20 microbial cultures (both standard and clinical strains) were used in this study. Nineteen strains of clinical MRSA isolates cultured from patient samples were kindly provided by Ramathibodi Hospital and Nakhon Pathom Hospital, Thailand. Pathogen purification and identification were confirmed using a microbial identification system at the Department of Microbiology, Faculty of Pharmacy, Mahidol University, Thailand. The standard strain of *S. aureus*,ATCC 25923 (methicillin−sensitive *Staphylococcus aureus*, MSSA), was obtained from the Thailand National Institutes of Health and was used as a control strain. 

The microorganisms were maintained in a mixture of tryptic soy broth (TSB; Becton Dickinson & Co., France) and 30% w/v glycerol at −80 °C until use. For experiments, all of the bacterial strains were grown separately on tryptic soy agar (TSA; Becton Dickinson & Co., France) at 37 °C for 18–24 h. The isolated bacterial colonies of actively growing cultures from agar plates were transferred to a test tube with TSB and incubated at 37 °C for 24 h in shaker. The culture turbidity was adjusted spectrophotometrically at 600 nm to obtain an optical density (OD) of 0.2 (approximately 10^6^–10^7^ CFU/mL) before use as an inoculum in the antimicrobial susceptibility test. 

### 3.2. Antimicrobial Susceptibility Test

#### 3.2.1. Disc Diffusion Method

The antimicrobial activity of the MSKE was screened for its inhibitory activity by the disc diffusion test following the procedures recommended by the Clinical and Laboratory Standards Institute (CLSI) with slight modifications [19]. Briefly, the following concentrations of MSKE were prepared by serial dilution in 10% aqueous DMSO: 500, 250, 125 and 62.5 mg/mL. Each prepared inoculum was swabbed on TSA and air−dried at room temperature (25 °C). A 6-mm sterile paper disc was loaded with 10 μL of the prepared MSKE solution, and the disc was placed on the agar plate. The plates were left to dry and then were incubated at 37 °C for 24 h under aerobic conditions. A negative control was prepared using the same solvents employed to dissolve the MSKE, and vancomycin (30 µg/disc) was used as a positive control. All disc diffusion tests were performed in triplicate, and the antibacterial activity was expressed as the mean of the inhibition diameter (mm).

#### 3.2.2. Determination of the MICs and MBCs

The broth microdilution method was used to determine the MIC of MSKE and its phenolic principles. Serial two–fold dilutions of test compounds were mixed with TSB at a 1:1 ratio (v/v) in 96-well sterile microtitre plates to obtain final concentrations of 0.06–3.75 mg/mL for MSKE, 0.02–1.00 mg/mL for PGG and 0.05–3.00 mg/mL for GA and MG. Then, 50 μL of a 1:5 dilution of the prepared inoculum was added to TSB supplemented with the test compounds to obtain a 100 μL final volume in each well. The microtitre plates were then incubated at 37 °C overnight under aerobic conditions. In each test, the following controls were used: (1) a negative control including the test sample but not the organism; and (2) a positive control without the test sample but containing the organism. Vancomycin and penicillin G were used as reference standards. The MIC was defined as the lowest concentration at which no bacterial growth was observed by the unaided eye. The amount of growth in the wells containing test samples was compared with the amount of growth in the control wells when determining the growth endpoints. To establish the MBC, 20 µL of each culture medium was removed from wells with no visible growth and inoculated on TSA plates. After aerobic incubation at 37 °C overnight, the number of surviving organisms was determined. The MBC was defined as the lowest concentration that produced a complete suppression of visible colony growth. Each sample was tested in triplicate in separate experiments. The enhancing effects of MSKE and its phenolic principles on the antibacterial activity of penicillin G were evaluated by the broth microdilution method. The MIC and MBC values of penicillin G were determined in combination with 0.25, 0.5 or 1 MIC of the test compounds.

#### 3.2.3. Time−Kill Assay

The bactericidal activities of MSKE and its phenolic principles were determined according to the time–kill assay of Chusri *et al.* [20] with slight modification. The bacterial suspension (100 µL, approximately 10^6^ CFU/mL) was added to TSB (900 µL) containing the test sample at 4 and 8 MICs. After incubation at 37 °C, sample (100 µL) was collected at different time intervals (0, 2, 4, 6, 8, 12, 16 and 24 h) and a ten–fold serial dilution was prepared in sterile saline (0.9% w/v NaCl). Thereafter, 25 µL of each dilution was placed on a TSA plate and incubated at 37 °C for 24 h. A count for viability was performed, and the number of CFU/mL was recorded. A bacterial growth control was included in each assay and consisted of 1% DMSO without the addition of test samples. Vancomycin was also used as the reference antibiotic. All experiments were carried out at least in duplicate. Time–kill curves were constructed by plotting log_10_ CFU/mL against time. Bactericidal activity was defined as a ≥3 log_10_–fold decrease in the number of survivors at each time point compared with the number inoculated at time zero. This activity was equivalent to 99.9% killing of the inoculum [9].

### 3.3. Electron Microscopy

#### 3.3.1. SEM

The prepared inoculum (200 µL) of *S. aureus* ATCC 25923 was transferred into TSB (1.8 mL) containing MSKE or PGG at a MIC of 4. Bacterial growth controls were performed with the addition of 1% DMSO without the test samples. After the suspensions were incubated at 37 °C for 12 h, bacterial cells were collected by centrifugation at 3,000 rev/min for 10 min. Samples were then fixed in 2.5% w/v of glutaraldehyde at 4 °C for at least 2 h. The cells were washed with 0.1 M phosphate buffer solution (PB, pH 7.2) and postfixed in 1% w/v osmium tetroxide in 0.1 M PB for 1–2 h. Cells were dehydrated using serial concentrations of ethanol (35, 50, 70, 95, and 100%). After critical point drying and coating with gold sputter, samples were examined using a Hitachi S501 scanning electron microscope. 

#### 3.3.2. TEM

The prepared inoculum (200 µL) of *S. aureus* ATCC 25923 and the M09 MRSA strain were transferred into TSB (1.8 mL) supplemented with MSKE at 1, 2 or 4 MICs. Bacterial growth controls were performed with the addition of 1% DMSO without the test samples. The suspensions were incubated at 37 °C for 12 h. The bacterial cells were then harvested by centrifugation at 3,000 rev/min for 10 min. The samples were fixed in 2.5% w/v of glutaraldehyde at 4 °C for at least 2 h and postfixed in 1% w/v osmium tetroxide in 0.1 M PB for 1–2 h. The bacterial cells were dehydrated using serial concentrations of ethanol (35, 50, 70, 95, and 100%) and embedded in Spurr’s resin. The samples were cut with an ultramicrotome (LKD, Sweden) and stained with uranyl acetate and lead citrate. The ultrathin sections were examined under a transmission electron microscope (JEM−200CX, JEM−2100, JEOL, Japan).

### 3.4. Statistical Analysis

All experimental results were expressed as mean ± standard deviation (S.D.). All statistical analyses were carried out using SPSS (version 16.0 for Windows). Analysis of variance was performed by ANOVA. Significant differences between the means were determined using Tukey’s pairwise comparison test at a significance level of *p* < 0.01.

## 4. Conclusions

MSKE and its phenolic principles exhibited potent inhibitory effects against the standard MSSA strain and clinical MRSA isolates. PGG, the major phenolic principle of MSKE, appeared to be the major contributor to the inhibitory potency of MSKE. Damaging effects on the cell membrane that led to the alteration in cell morphology and interference with bacterial division were possible inhibitory mechanisms. MSKE at 0.25 and 0.5 MICs displayed a remarkable capacity to enhance the antibacterial activity of penicillin G by lowering the MIC of penicillin G by 10−20 fold. Despite the weak antimicrobial effects of MG against MRSA and MSSA, MG demonstrated the capacity to enhance the bacterial susceptibility to penicillin G of approximately 254−fold. Together, these results indicate that MSKE may potentially be useful as an alternative natural therapeutic agent or as an adjunctive therapy along with penicillin G against MRSA infections.

## References

[1] Stapleton P.D., Taylor P.W. (2002). Methicillin resistance in *Staphylococcus aureus*: Mechanisms and modulation. Sci. Prog..

[2] Marin M. (2002). Methicillin resistant Staphylococcus. Medicina (B Aires).

[3] Hiramatsu K. (2001). Vancomycin-resistant *Staphylococcus aureus*: A new model of antibiotic resistance. Lancet Infect. Dis..

[4] Gibbons S. (2004). Anti-staphylococcal plant natural products. Nat. Prod. Rep..

[5] Nithitanakool S., Pithayanukul P., Bavovada R. (2009). Antioxidant and hepatoprotective activities of Thai mango seed kernel extract. Planta Med..

[6] Nithitanakool S., Pithayanukul P., Bavovada R., Saparpakorn P. (2009). Molecular docking studies and anti-tyrosinase activity of Thai mango seed kernel extract. Molecules.

[7] Leanpolchareanchai J., Pithayanukul P., Bavovada R., Saparpakorn P. (2009). Molecular docking studies and anti-enzymatic activities of Thai mango seed kernel extract against snake venoms. Molecules.

[8] Pithayanukul P., Leanpolchareanchai J., Saparpakorn P. (2009). Molecular docking studies and anti-snake venom metalloproteinase activity of Thai mango seed kernel extract. Molecules.

[9] Verma P., Schwalbe R., Steele-Moore L., Goodwin A.C. (2007). Methods for determining bactericidal activity and antimicrobial interactions: Synergy testing, time-kill curves, and population analysis. Antimicrobial susceptibility testing protocols.

[10] Gadhi C.A., Hatier R., Mory F., Marchal L., Weber M., Benharref A., Jana M., Lozniewski A. (2001). Bactericidal properties of the chloroform fraction from rhizomes of *Aristolochia paucinervis* Pomel. J. Ethnopharmacol..

[11] Chusri S., Voravuthikunchai S.P. (2009). Detailed studies on *Quercus infectoria* Olivier (nutgalls) as an alternative treatment for methicillin-resistant *Staphylococcus aureus* infections. J. Appl. Microbiol..

[12] Hamilton-Miller J.M.T., Shah S. (1999). Disorganization of cell division of methicillin-resistant *Staphylococcus aureus* by a component of tea (*Camellia sinensis*): A study by electron microscopy. FEMS Microbiol. Lett..

[13] Haslam E. (1996). Natural polyphenols (vegetable tannins) as drugs: Possible modes of action. J. Nat. Prod..

[14] Scalbert A. (1991). Antimicrobial properties of tannins. Phytochemistry.

[15] Koren E., Kohen R., Ovadia H., Ginsburg I. (2009). Bacteria coated by polyphenols acquire potent oxidant−scavenging capacities. Exp. Biol. Med..

[16] Yi S.M., Zhu J.L., Fu L.L., Li J.R. (2010). Tea polyphenols inhibit *Pseudomonas aeruginosa* through damage to the cell membrane. Int. J. Food Microbiol..

[17] Shibata H., Kondo K., Katsuyama R., Kawazoe K., Sato Y., Murakami K., Takaishi Y., Arakaki N., Higuti T. (2005). Alkyl gallates, intensifiers of β-lactam susceptibility in methicillin-resistant *Staphylococcus aureus*. Antimicrob. Agents Chemother..

[18] Shiota S., Shimizu M., Sugiyama J., Morita Y., Mizushima T., Tsuchiya T. (2004). Mechanisms of action of corilagin and tellimagrandin I that remarkably potentiate the activity of β-lactams against methicillin-resistant *Staphylococcus aureus*. Microbiol. Immunol..

[19] Clinical and Laboratory Standards Institute (2006). Performance Standards for Antimicrobial Disk Susceptibility Tests: Approved Standard M2-A9.

[20] Chusri S., Voravuthikunchai S.P. (2008). *Quercus infectoria*: A candidate for the control of methicillin-resistant *Staphylococcus aureus* Infections. Phytother. Res..

